# Laws for Stamping out Cattle-Tuberculosis in France

**Published:** 1896-06

**Authors:** 


Laws for Stamping Out Cattle-tuberculosis in France. A bill which aims at stamping out cattle-tuberculosis in France is now before the French parliament. The chief regulations are the following: All cattle which present the clinical symptoms of tuberculosis are to be killed immediately. The prefect of the district shall issue the order for slaughter. Cattle whose clinical symptoms awake suspicion that they may be sick with tuberculosis are to be subjected to the tuberculin-test. The animals which react after this test are to be killed. If tuberculosis is established in a living, slaughtered, or dead animal, all the cattle which have been with it in the same pen or pasture are to be subjected to the tuberculin-test. The animals which react are to be sold, and are to be handed over to the butcher within the period of one year. This period, on report of the Committee on Epidemics, can be prolonged by the Minister of Agriculture, but the owner then loses all right to indemnification. Every animal which reacts upon the tuberculin-test and which shows the clinical phenomena of tuberculosis during the period of observation is to be killed immediately. In the case of complete or partial confiscation of the flesh of tuberculous animals, the owners, if the killing is due to this enforcement of the preceding regulations, shall receive the following indemnification: 1. One-fourth of the value of the meat confiscated if the animal has been killed in consequence of an order from the prefect. 2. The half of the value of the meat confiscated if the animal, in accordance with the regulations of Art. 3, has been handed over to the butcher within the period of one year, and showed no clinical symptoms of tuberculosis. The confiscation of meat in every other case gives no right to indemnification.—*Ibid.*

